# Editorial: Nuclear Receptors and Coregulators in Metabolism and Immunity

**DOI:** 10.3389/fendo.2021.828635

**Published:** 2021-12-24

**Authors:** Rongrong Fan, Ines Pineda-Torra, Nicolas Venteclef

**Affiliations:** ^1^ Department of Biosciences and Nutrition, Karolinska Institutet (KI), Solna, Sweden; ^2^ Centre for Cardiometabolic and Vascular Science, Department of Medicine, University College London, London, United Kingdom; ^3^ INSERM, Cordeliers Research Centre, Sorbonne Paris Cité, Université Paris Descartes, Université Paris Diderot, Paris, France

**Keywords:** nuclear receptor, coregulator, obesity, inflammation, immune cells, NAFLD, obesity, multiple sclerosis

Dysregulated tissue metabolism and inflammation are associated with many human diseases such as metabolic disorders, autoimmune diseases and cancer (1, 2). The transcriptional alterations in both metabolic and immune cells in response to microenvironment-derived pathological stimulus are mostly linked with abnormalities of transcription factors (TFs). Nuclear receptors (NRs) are a family of ligand-dependent TFs. For most of them, their activities can be controlled by both endogenous and exogenous molecules such as metabolites, steroid hormones and synthesized chemicals (3). As a result, NRs have been appealing drug targets for many decades, with already approved compounds with promising therapeutic outcomes.

One of such NRs is the Peroxisome Proliferator-Activated Receptor γ (PPARγ). PPARγ is among the most extensively studied NRs. It is well-known as the master regulator of adipose tissue biology (4). Using an *in vitro* 3T3L1 adipocyte cell model, Dias et al found that PPARγ phosphorylation at serine 273 (S273) by cyclin-dependent kinase 5 (CDK5) caused a coactivator-to-corepressor switch and thereby decreased PPARγ activities and reduced mRNA expression of metabolically protective adipokines. In addition to adipocytes, many studies have discovered that PPARγ have pleiotropic functions in various other cell types and tissues, including colon, breast, prostate and bladder, as well as immune cells such as monocytes/macrophages, dendritic cells and lymphocytes. The multi-organ functions, dysregulations (mRNA expression changes, gain or loss of functional mutations, etc.) and molecular mechanisms underlying PPARγ activities are summarized by Hernandez-Quiles et al.

Another well-studied NR is glucocorticoid receptor (GR). GR is an important regulator of many physiological processes (5). Due to the strong anti-inflammatory function of GR, its agonists have been widely applied in the clinic for severe immune diseases. The usage of GR agonists is limited by the side effects including severe responses in key metabolic organs such as liver. Ongoing efforts aim to: 1) understand the regulatory mechanisms of GR, i.e. the coregulatory factors and complexes, functionality of different isoforms, in major metabolic organs such as liver, which is reviewed by Præstholm et al; and 2) identify so-called selective GR modulators (SGRM) with more tissue- or isoform-specificity in order to minimize the unwanted metabolic effects of GR, which has not been successful so far. Van Moortel et al. discussed the bottlenecks of pharmaceutical discovery of better GR ligands with emphasis on both ongoing research developments and potential solutions.

A classic paradigm of NR biology relies on lipid-sensing NRs as hubs to connect metabolism and inflammation. Lipids such as cholesterols play crucial roles in physiology and thus are tightly regulated by multiple NRs, including farnesoid X receptors (FXRs) and liver X receptors (LXRs). Johansson et al. investigated the hepatic FXR/fibroblast growth factor 19 (FGF19) axis in cholesterol excretion as bile acids (BAs). FGF19 is derived from both liver and intestine in response to BAs and is believed to be essential for the FXR to inhibit BA synthesis. The FXR/FGF19 connection was studied in primary human hepatocytes. Despite that FXR activation upregulated FGF19 secretion in the hepatocytes, FXR did not require FGF19 to inhibit BA synthetic genes. The authors therefore proposed independent regulatory roles of FXR and FGF19 in human liver BA production.

Overload of lipids (especially cholesterol) is related to many human diseases. In macrophages, accumulation of cholesterol causes inflammation and plaque development in atherosclerosis (6). Ramírez et al. discovered that ligand activation of LXR induced caveolin-1 expression. Because caveolin-1 is responsible for the formation of caveolae, multi-functional lipid raft microdomains of the membrane with high concentrations of cholesterol, LXR-induced caveolin-1 eliminated cholesterol in macrophages and alleviated atherosclerosis. Excessive cholesterol is also involved in the progression of multiple sclerosis (MS). MS is an autoimmune disease caused by constitutively activated immune cells in the brain. Systemic changes of cholesterol and oxysterol may contribute to the disease by modulating the activities of LXRs, and thereby causing immune cell dysregulation in the human MS development. The interplay between cholesterol, oxysterols and LXRs, as well as the potential therapeutic application of LXR agonists in human MS pathology have been reviewed by Pineda-Torra et al. in this Research Topic.

At the molecular level, genome-wide analysis of lipid-sensing NR binding with next generation sequencing (NGS) has revealed a major portion of the NR cistromes that are not responsive to ligand activation. In tissue macrophages, the lipid-sensing NRs work as lineage determining TFs (LDTFs) to define macrophage subsets. They can also recruit coregulators independent of ligand binding to regulate the epigenetic remodeling and 3D structure of chromatin. The new concept of the non-classic or ‘unorthodox action’ of lipid-sensing NRs are reviewed by Czimmerer et al.

The NRs also participate in innate immunity *via* a crosstalk with inflammasome pathways in the macrophages. Several NRs have been reported to work with inflammatory TFs such as NFκB to control inflammasome priming by regulating its component gene expression. NRs such as FXRs can physically interact with the NLRP3 and caspase1 to directly inhibit the complex assembly and the enzymatic activities. On the other hand, activated inflammasome also modulates NRs, i.e. by directly cleaving the NRs at conserved cleavage sites. The NR/inflammasome interaction is involved in multiple diseases, which is systemically reviewed by Duez and Pourcet. Beyond that, NRs are important regulators of adapted immune responses by regulating T cells. The NR4A family of orphan nuclear receptors (receptors with unrecognized ligands) not only controls T cell differentiation and development, but also defines the acute and chronic responses of CD4+ and CD8+ T cells. The underlying mechanisms of NR4A-mediated adaptive immune regulation were reviewed by Odagiu et al. in this Research Topic.

It has long been observed that metabolic and inflammatory responses differ between males and females. This is partially attributed to sex-specific steroid hormones that act as ligands of NRs such as estrogen receptors (ERs). Among the metabolic organs, liver shows the highest degree of sexual dimorphism. This aligns with the regulation of ER in both metabolic and inflammatory pathways in the liver, which is summarized by Della Torre in this Research Topic. Fluctuation of ER activities during physiological and pathological conditions leads to altered functions in both metabolic organs and immune cells and is linked with diseases such as breast cancer. Brundin et al. investigated the expression of ER subtypes in different cell subsets of human peripheral blood mononuclear cells (PBMCs), and their correlation with multiple inflammatory genes. The study confirmed the association of ER dysregulation with altered inflammation in PBMC cells during the menstrual cycle. Estradiol signaling through ER plays crucial roles in breast cancer cell development. Cervantes-Badillo et al. investigated the interaction between regulatory components of ER activities and identified the interferon alpha inducible protein 27 (IFI27/ISG12). IFI27/ISG12 could be induced by both interferon and estradiol in breast cancer cells. The protein then facilitated the interaction of ER with CRM1/XPO1 which retained ER in the cytoplasm and impaired its activities. As a result, IFI27/ISG12 elevation was associated with reduced overall survival of ER^+^ breast cancer patients and resistance to tamoxifen treatment.

There are still many challenges remaining in the NR research field despite the huge efforts invested. Further studies are required for better understanding of the molecular events regulated by NRs in different tissues. Such information will be of great value to develop ligands or NR-targeted therapeutic strategies with more specificity.

## Author Contributions

All listed authors have made equally substantial contribution to the editorial work of this Research Topic, both intellectually and physically.

## Funding

The work is supported by BRC funding (grant no: BRC792/CM/IPT/101320) from the NIHR University College London Hospitals Biomedical Research Centre, Swedish Research Council (Vetenskapsrådet, grant no: 2019-01884), Novo Nordisk Foundation (grant no: NNF19OC0056615), Region Stockholm's CIMED Foundation (grant no: 20190172), Åke Wibergs Stiftelse (grant no: M20-0001) and Swedish Cancer Foundation (Cancerfonden, grant no: 20 0680 Pj 01 H).

## Conflict of Interest

The authors declare that the research was conducted in the absence of any commercial or financial relationships that could be construed as a potential conflict of interest.

## Publisher’s Note

All claims expressed in this article are solely those of the authors and do not necessarily represent those of their affiliated organizations, or those of the publisher, the editors and the reviewers. Any product that may be evaluated in this article, or claim that may be made by its manufacturer, is not guaranteed or endorsed by the publisher.
